# Metabolic profile of complete spinal cord injury in pons and cerebellum: A 3T 1H MRS study

**DOI:** 10.1038/s41598-023-34326-1

**Published:** 2023-05-04

**Authors:** Johannes K. Richter, Vanessa Vallesi, Niklaus Zölch, Kimberly L. Chan, Nadine Hunkeler, Mihael Abramovic, Claus Hashagen, Ernst Christiaanse, Ganesh Shetty, Rajeev K. Verma, Markus F. Berger, Angela Frotzler, Heidrun Eisenlohr, Inge Eriks Hoogland, Anke Scheel-Sailer, Patrik O. Wyss

**Affiliations:** 1 Department of Radiology, Swiss Paraplegic Centre, Guido A. Zaech – Strasse 1, 6207 Nottwil, Switzerland; 2 Department of Diagnostic, Interventional, and Pediatric Radiology, University Hospital of Bern, Inselspital, University of Bern, Bern, Switzerland; 3 Institute of Radiology and Nuclear Medicine, GZO Hospital Wetzikon, Wetzikon, Switzerland; 4 Support Center for Advanced Neuroimaging (SCAN), Neuroradiology, University Hospital of Bern, Inselspital, Bern, Switzerland; 5 Swiss Paraplegic Research, Nottwil, Switzerland; 6 Department of Forensic Medicine and Imaging, Institute of Forensic Medicine, University of Zurich, Zurich, Switzerland; 7 Department of Psychiatry, Psychotherapy and Psychosomatics, Psychiatric Hospital, University of Zurich, Zurich, Switzerland; 8 Advanced Imaging Research Center, UT Southwestern Medical Center, Dallas, TX USA; 9 Image Science Institute, University Medical Center Utrecht, Utrecht, The Netherlands; 10 Clinical Trial Unit, Swiss Paraplegic Centre, Nottwil, Switzerland; 11 Digital Trial Intervention Platform, ETH Zurich, Zurich, Switzerland; 12 Outpatient Care Unit, Swiss Paraplegic Centre, Nottwil, Switzerland; 13 Department of Paraplegia, Rehabilitation and Quality Management, Swiss Paraplegic Centre, Nottwil, Switzerland; 14 Department of Neuroradiology, University Hospital Zurich, Zurich, Switzerland

**Keywords:** Neuroscience, Trauma

## Abstract

The aim of this exploratory study was the assessment of the metabolic profiles of persons with complete spinal cord injury (SCI) in three region-of-interests (pons, cerebellar vermis, and cerebellar hemisphere), with magnetic resonance spectroscopy, and their correlations to clinical scores. Group differences and association between metabolic and clinical scores were examined. Fifteen people with chronic SCI (cSCI), five people with subacute SCI (sSCI) and fourteen healthy controls were included. Group comparison between cSCI and HC showed lower total N-acetyl-aspartate (tNAA) in the pons (*p* = 0.04) and higher glutathione (GSH) in the cerebellar vermis (*p* = 0.02). Choline levels in the cerebellar hemisphere were different between cSCI and HC (*p* = 0.02) and sSCI and HC (*p* = 0.02). A correlation was reported for choline containing compounds (tCho) to clinical scores in the pons (rho = − 0.55, *p* = 0.01). tNAA to total creatine (tNAA/tCr ratio) correlated to clinical scores in the cerebellar vermis (rho = 0.61, *p* = 0.004) and GSH correlated to the independence score in the cerebellar hemisphere (rho = 0.56, *p* = 0.01). The correlation of tNAA, tCr, tCho and GSH to clinical scores might be indicators on how well the CNS copes with the post-traumatic remodeling and might be further examined as outcome markers.

## Introduction

Spinal cord injury (SCI) causes loss of muscle function, sensation and autonomic function. Studies have shown that changes do not only occur at the level of injury in the spinal cord, but also above and below that level in the spinal cord in addition to alterations in the brain^1,2^. Morphometric reorganization and metabolic alterations of the motor cortex have been described^3,4^. Chronic brain neuro-inflammation with concomitant neurodegeneration and cognitive decline have been observed in rodents^5^ and similar degenerative changes have been showed in humans^6^.

The cerebellum plays an important role in movement control. In particular, the cerebellar vermis receives information from the sensory system (e.g. non-conscious proprioception) via the spinocerebellar tract and—among other functions—coordinates and modulates motor response^7^. It is essential for a wide array of brain functions, including posture and locomotion^8^.

Metabolic changes within the cerebellum and vermis have been reported in people with ataxia^9^. Animal studies with rats suggest changes in these regions in SCI^10^. Therefore, the aim of this study was to examine cerebellar metabolic profiles in SCI in humans by means of MR Spectroscopy, which is a non-invasive method to determine alterations within the brain regarding neuronal metabolism.

In a recently published study, we showed that the levels of glutathione (GSH) in the pons (acquired with standard point-resolved spatially localized spectroscopy (PRESS) sequence) correlated to the motor score improvements during rehabilitation in people with **in**complete SCI (i.e. American spinal injury association Impairment Scale grade B, C, and D; AIS B, AIS C and AIS D)^11^.

However, this exploratory study evaluated the metabolic profile within the pons, the cerebellar vermis, and cerebellar hemispheres in people with complete (AIS A) chronic (injury > 2 years) and subacute (injury < 28 weeks) SCI. Furthermore, the advanced metabolite cycling (MC) technique was applied to acquire spectra.

Our working hypothesis presumed that total N-Acetyl-Aspartate (tNAA), reflecting neuron integrity, will show a positive correlation with the clinical neurological scores during recovery from neuronal injury. NAA has been reported as marker for stroke recovery^12^. With GSH on the other hand, being a major antioxidant and as such a neuroprotective agent, we expected that patients with high GSH levels might perform better^11^. Finally, choline containing compounds (tCho) as membrane turnover and neuro-inflammation marker was expected to be correlated with poorer scores (which has been shown in traumatic brain injury)^13,14^, and was expected to be generally low in the late stages of SCI. With total creatine (tCr) being an indicator for neuronal tissue energetics, we were particularly interested in the tNAA/tCr ratio, reflecting tissue health vs. metabolic activity. People with complete SCI were chosen as in this patient group the largest fluctuations in metabolite concentrations both immediately after SCI as well as during rehabilitation was expected^13,15^, as compared with people with lower degrees of spinal injury. From a clinical perspective, looking to establish an MRS surrogate marker for the potential of neurological rehabilitation in the patients most gravely affected from spinal injury seemed a priority over predictors of rehabilitation outcome in patients with lesser spinal injuries.

## Results

### Demographics

All study participants completed the full protocol, i.e. the MRI examination and clinical assessments. Two participants with SCI (one subacute, one chronic) had to be excluded due to high Hospital Anxiety and Depression Scale (HADS) score (> 7). We included 15 participants with a chronic (> 2 years) complete (AIS A) injury (cSCI, median age: 58 years, IQR range: 45–62 years; time since injury: median = 19, range: 11–35 years, 2 women), 5 participants with a subacute (< 28 weeks after injury) complete (AIS A) spinal cord injury (sSCI, median age: 41 years, IQR range: 38–47 years; years since injury: median = 12 weeks, range: 8–16 weeks, 3 women), and 14 healthy control participants (HC, median age: 40 years, range: 31–54 years, 6 women). There was a significant difference in age but not gender between the groups (*p* < 0.001 and *p* = 0.2, resp.). The demographic data are summarized in Table 1.

**Table 1:** Demographics of the study participants.

Demographics	Chronic SCI (cSCI)	Subacute SCI (sSCI)	Healthy controls (HC)
Number of participants (n)	15	5	14
Gender: female/male	2/13	3/2	6/8
Median age, IQR range [y]	58; 45–62	41; 38–47	40; 31–54
Mean age ± SD [y]	54.2 ± 11.2	45.1 ± 12.8	42.5 ± 13.5
Full range: min–max [y]	32–70	32–68	26–64
Time since injury:			N/A
Median, IQR range [y]	19.0; 11–35	0.24; 0.16–0.31	
Mean ± SD [y]	21.9 ± 13.3	0.24 ± 0.08	
Full range: min–max [y]	5–50	0.14–0.35	
Neurological Level of Injury	Th2: 1	Th3: 1	N/A
	Th3: 1	Th4: 1	
	Th4: 3	Th6: 1	
	Th5: 1	Th11: 1	
	Th6: 4	Th12: 1	
	Th9: 1		
	Th10: 3		
	Th11: 1		

### Quality of MR spectroscopy measurements

**Figure 1:**
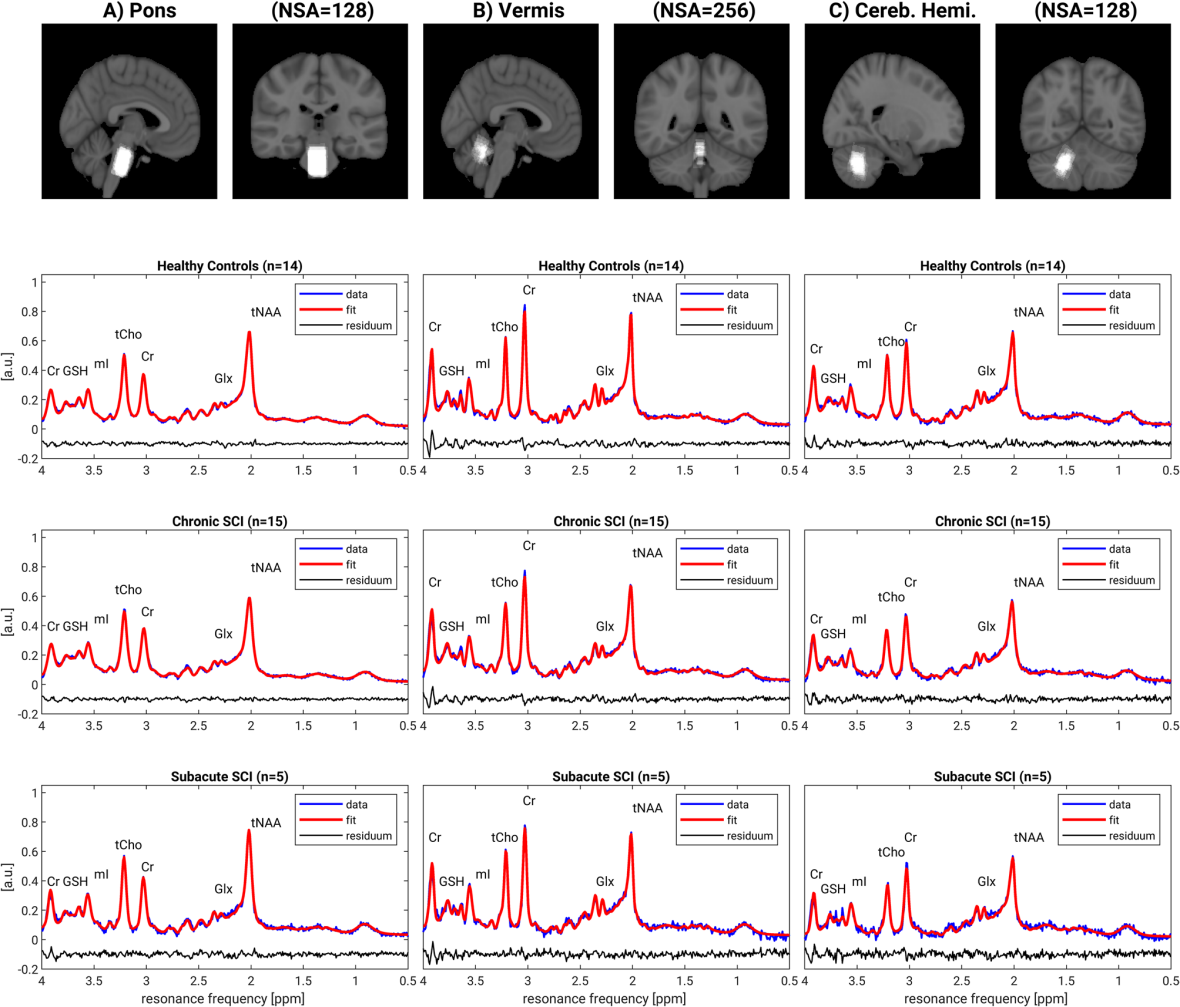
**Overview of voxel localization and spectra**. Illustration of the spectroscopic voxels of interest (top row) acquired in the pons (**A**), the cerebellar vermis (**B**) and the cerebellar hemisphere (**C**). For visualization purposes, all spectra acquired in every subgroup are frequency aligned to the water reference signal acquired with metabolite cycling technique and then averaged. The averaged spectrum is fitted and the data (blue), the fit (red) and the residuum (black) is shown for healthy controls (second row, number of subjects n = 14), participants with chronic SCI (third row, n = 15), and participants with subacute SCI (last row, n = 5). However, in the analysis process, the spectra from each individual are examined individually.

Figure 1 illustrates the overlay of all acquired spectroscopic voxels of interest (top row) acquired in the pons (A), the cerebellar vermis (B) and the cerebellar hemisphere (C). All spectra acquired in every subgroup are frequency aligned to the water reference signal acquired with MC technique and then averaged. The averaged spectrum is fitted and the data, the fit and the residuum is shown for healthy controls (second row, number of subjects n = 14), participants with chronic SCI (third row, n = 15), and participants with subacute SCI (last row, n = 5). The median signal-to-noise ratio (SNR) in the pons was highest (cSCI: 23; sSCI: 22, HC: 26), in-between in the cerebellar vermis (cSCI: 17; sSCI: 18; HC: 19) and lowest in the cerebellar hemisphere (cSCI: 13; sSCI: 13, HC: 14). The median of the full-width at half maximum had ordinary values between 5 and 7 Hz for the three regions, indicating good overall spectral quality for in-vivo MRS. The Cramer-Rao Lower Bound (CRLB) of NAA served as postprocessing quality measures with values between 2 and 3 for the pons and cerebellar vermis regions and values between 3 and 6 for the cerebellar hemisphere region of interest.

### Metabolic changes in the pons, the cerebellar vermis and the cerebellar hemisphere

**Figure 2:**
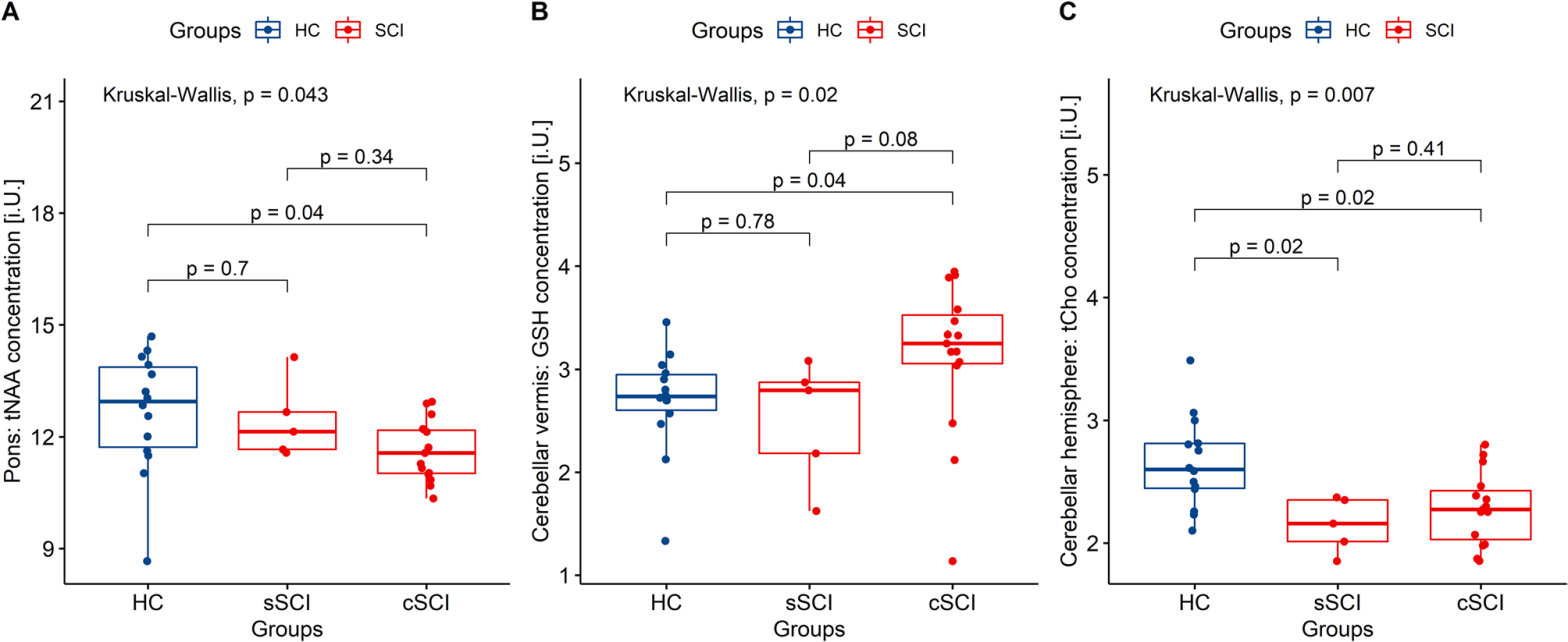
**Group differences**. Significant group differences plots. Total N-acetyl-aspartate (tNAA) in the pons (**A**), glutathione (GSH) in the cerebellar vermis (**B**) and choline containing compounds (tCho) in the cerebellar hemisphere.

We found group differences for the following metabolites (Fig. 2, Supplementary Table S1). In the pons, we report group differences in tNAA (*p* = 0.04, Bayes Factor BF_10_ = 1.42). Post-hoc analysis showed significant differences in the concentration of tNAA between participants with chronic SCI and HC (*p* = 0.04, BF_10_ = 2.15). In the cerebellar vermis, we report group differences for GSH (*p* = 0.02, BF_10_ = 1.12): Post-hoc tests showed differences between participants with chronic SCI and HC (*p* = 0.04, BF_10_ = 1.19). Finally, in the cerebellar hemisphere, we report different tCho concentration between the groups (*p* = 0.01, BF_10_ = 12.76). Post-hoc analysis showed a significant difference between the level of HC to participants with both chronic (*p* = 0.02, BF_10_ = 7.24) and subacute (*p* = 0.02, BF_10_ = 4.46) SCI.

### Metabolic concentration and clinical scores

**Figure 3:**
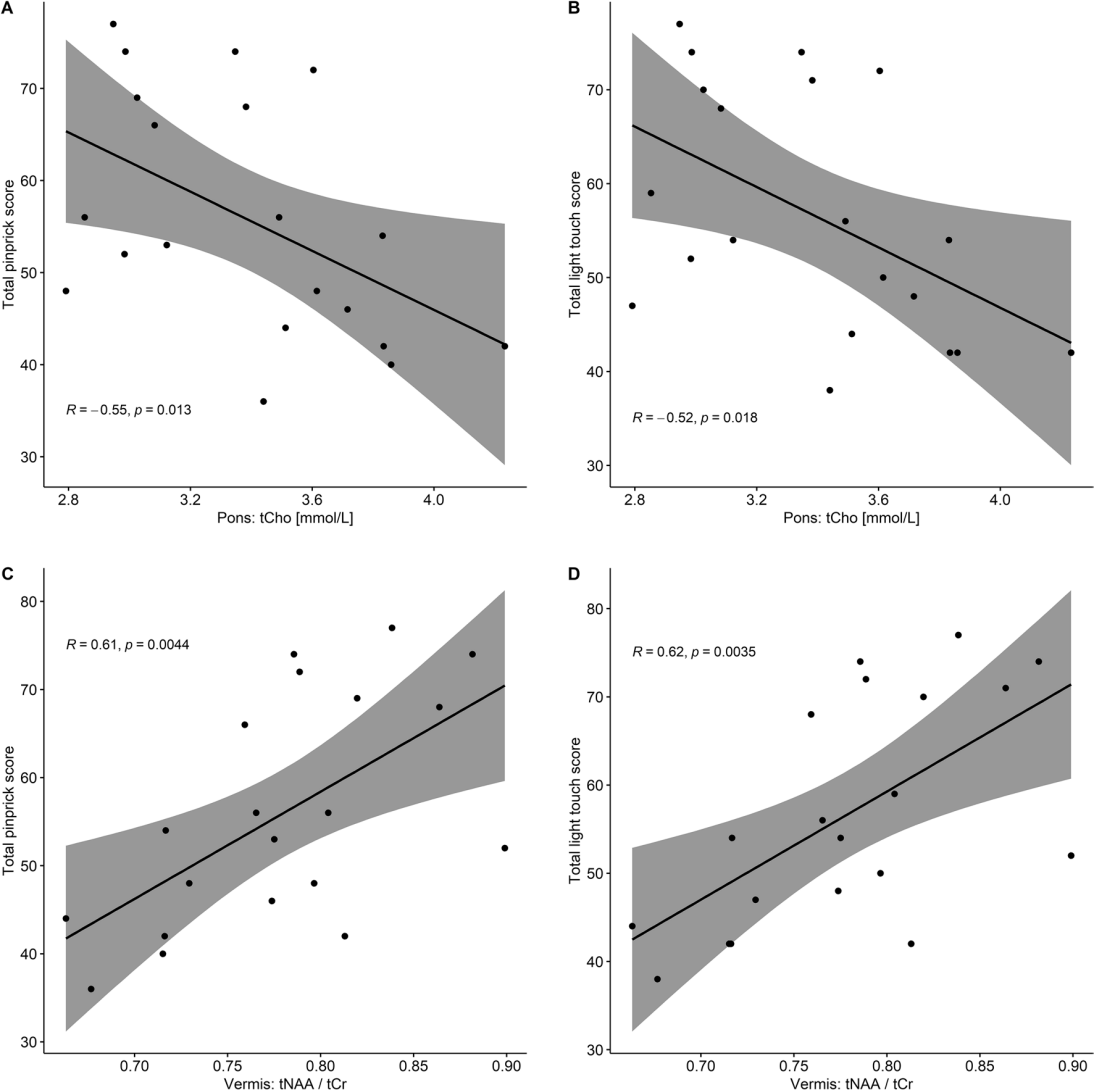
**Correlation of metabolites to pinprick and light touch scores**. Correlation of the metabolites to light touch and pinprick scores (American spinal injury association clinical scores). (**A**, **B**) Total choline (tCho) concentration in the pons is negatively correlated to total pinprick and total light-touch scores, resp. (**C**, **D**) Ratio of total NAA to total creatine (tNAA/tCr) in the cerebellar vermis is positively correlated to total pinprick and total light-touch score.

We examined the correlation between the metabolite concentrations and the clinical scores (LT: Light touch; PP: pinprick; SCIM: Spinal Cord Independence Measure). We report a negative correlation of tCho to PP (rho = − 0.55, *p* = 0.01, BF_10_ = 3.23) and LT (rho = − 0.52, *p* = 0.02, BF_10_ = 3.32) scores in the pons, and a positive correlation of the ratio of tNAA/tCr to PP (rho = 0.61, *p* = 0.004, BF_10_ = 10.30) and LT (rho = 0.62, *p* = 0.004, BF_10_ = 11.39) in the cerebellar vermis (Fig. 3, Supplementary Table S2).

**Figure 4:**
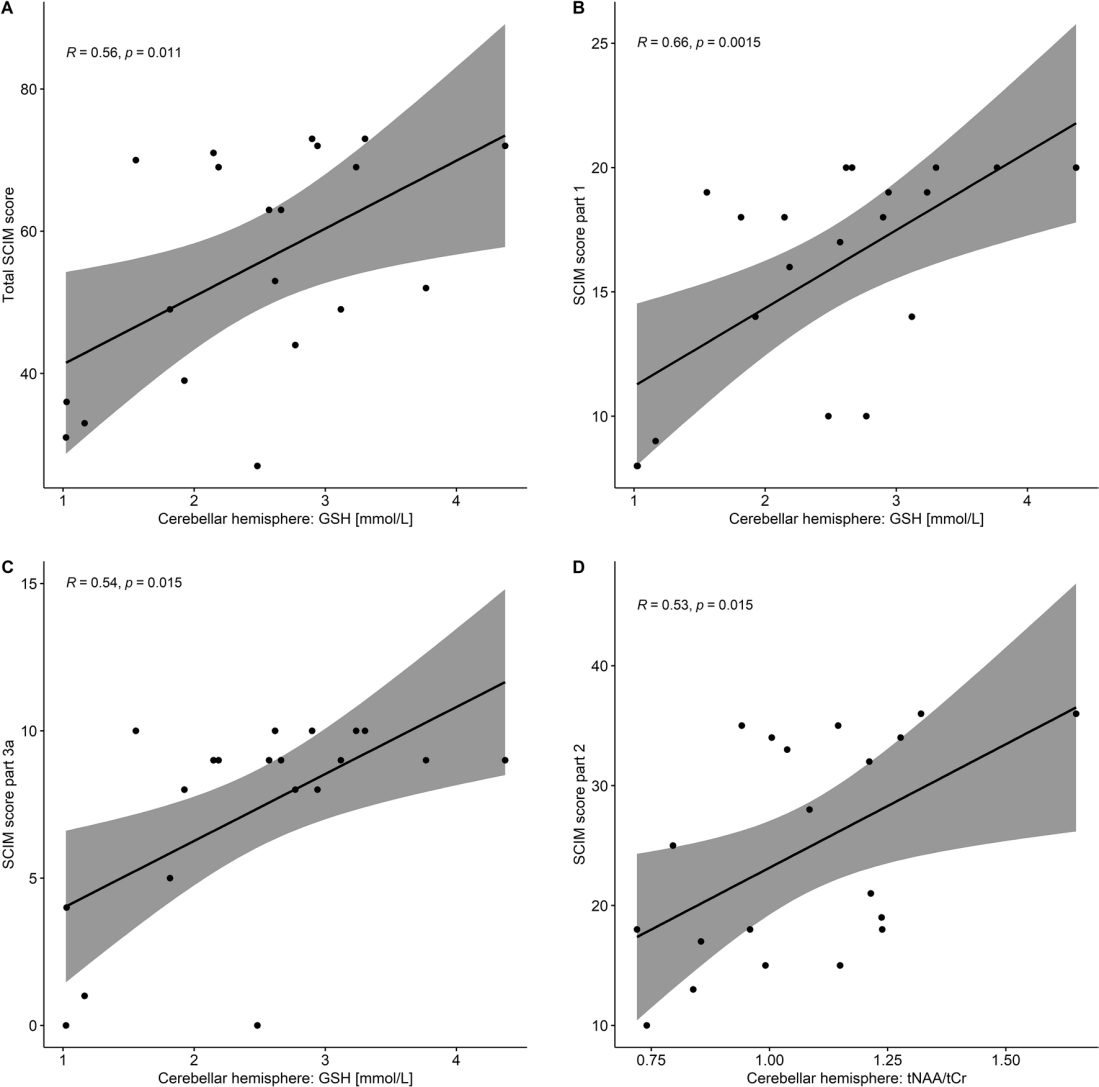
**Correlation of metabolites to spinal cord independence measures.** Correlation of the metabolites in the cerebellar hemisphere to spinal cord independence measure (SCIM). The level of glutathione (GSH) is positively related to the total SCIM score (**A**), part 1 (self-care) (**B**) and part 3a (indoor mobility) (**C**). Creatine is negatively correlated to the SCIM part 2 scores (**D**).

For SCIM score, we found significant correlations in the cerebellar hemisphere: We report a positive correlation of GSH to the total SCIM score (rho = 0.56, *p* = 0.01, BF_10_ = 4.66), SCIM score part 1 (rho = 0.66, *p* = 0.002, BF_10_ = 15.00) and SCIM part 3a (rho = 0.54, *p* = 0.02, BF_10_ = 9.64). We report a negative correlation between tCr and SCIM part 2 (rho = − 0.46, *p* = 0.04, BF_10_ = 2.41) and a positive correlation between tNAA/tCr and SCIM part 2 (rho = 0.53, *p* = 0.02, BF_10_ = 4.22) (Fig. 4, Supplementary Table S2).

## Discussion

We conducted a prospective and exploratory clinical observation study in people with paraplegic complete SCI (AIS A) including two clinical groups (subacute and chronic).

SNR was sufficient and data were acquired in the pons, the cerebellar vermis and in the right side of the cerebellar hemisphere. However, the data quality was lowest in the cerebellar hemisphere, which was expected due to the complex microstructural architecture of the cerebellar hemisphere.

We reported group differences in the metabolite concentration between the three groups (cSCI, sSCI and HC) and showed correlations between metabolites in different ROIs and clinical scores.

The lower tNAA in chronic SCI is expected, as tNAA is reduced in most diseases or degenerative processes^16^, indicating less neurons per volume unit. Boesch et al. showed reduced tNAA/tCr e.g. in people with ataxia^17^. The tNAA/tCr ratio in subacute SCI might indicate ongoing transition from the tNAA/tCr levels of HC to those of cSCI.

The level of GSH is higher in chronic SCI as compared to HC and sSCI. This might be due to increased oxidative stress^18^. Higher GSH levels might indicate a better adaptation, possibly resulting in better clinical outcome.

Total choline (tCho) is significantly lower in participants with SCI indicating an effect of Wallerian degeneration beginning within the first days after injury, and, depending on the imaging method, has been reported to progress within weeks to months after imaging^19,20^. The concentration levels of the subacute and chronic SCI do not differ and the tCho concentration is lower than in HC. A possible explanation might be the reduced membrane turnover as precursor of neurodegeneration^21^.

The similar level of tCho in both SCI groups might indicate a different time scale applied for tCho compared to effects influencing the levels of tNAA and GSH.

In a recently published study, we showed that GSH correlated to motor score in incomplete SCI during rehabilitation. However, in this study, we only examined pinprick and light touch scores from the ISNCSCI assessment; since the motor score is the same in all participants, i.e. people with complete paraplegic SCI all have a motor score of 50 out of 100, indicating full motor strength in the upper body part and no motor strength in the lower extremities. PP and LT represent different aspect in perception of pain and touch^22^. We report a negative correlation between tCho and PP/LT (i.e. the lower the tCho level, the higher the clinical score). A higher level of tCho possibly indicates a higher level of inflammation, which in turn might be disadvantageous for clinical improvements. On the other hand, the higher the level of tNAA/tCr indicating more viable neurons, the better the clinical score, confirming the finding of a positive correlation between tNAA/tCr in the cerebellar vermis and PP/LT.

Interestingly, we found a positive correlation between the level of GSH in the cerebellar hemisphere to total SCIM score and SCIM part 1 (self-care) and 3a (bed mobility and transfers), the scores ranging from 0 to 100 (total SCIM), 0–20 (part 1) and 0–10 (part 3a). The correlation of GSH in the cerebellar hemisphere to the total SCIM score might be explained by the anti-oxidative effect, thus higher GSH levels reflect a state of higher alertness against oxidative stress and therefore better clinical performance. Finally, the positive correlation of tNAA/tCr as shown in Fig. 4D and thus the negative correlation of tCr to SCIM part 2 (sphincter management) might be explained by a possible overcompensation, i.e. higher activity of neurons needed to achieve previous abilities. In summary, the higher GSH and tNAA and the lower tCr and tCho, the better the clinical outcome in people with complete paraplegic SCI. Further study might examine these biomarkers as possible therapy outcome measurement to improve rehabilitation therapy.

Our study had important limitations: The overall number of participants was small, especially in the subacute group (with only five valid data sets). However, we conducted non-parametric group comparison tests and used non-parametric correlation analysis to address this point. In addition to frequentist statistics (null hypothesis significance testing), we included Bayesian statistics to quantify the evidence from the data itself for or against the null hypothesis as has been previously described^11^, which does not require correction for multiple testing.

We acquired spectra in three distinct regions of interest with single voxel spectroscopy using the advanced MC technique. A future study might apply multi-voxel techniques to further investigate the effect of complete paraplegic SCI.

In conclusion, we showed group differences for the metabolites tNAA, GSH and tCho in data acquired in complete, paraplegic SCI and HC with means of short-echo MR spectroscopy. In addition, tNAA, tCr and tCho correlated with the pinprick and light touch scores in the pons and cerebellar vermis. Furthermore, GSH in the cerebellar hemisphere correlated with the SCIM score, which measures patients’ independence. Further studies might include complete tetraplegic patients to examine the influence of the level of injury on the degree of metabolite-alteration in the cerebellar and pontine regions.

## Materials and methods

### Institutional Review Board Approval

The local ethics committee northwest and central Switzerland (EKNZ) approved the following study (2019-01624), which was conducted in accordance with the Declaration of Helsinki. All participants were informed about the aim and procedure of this prospective study and gave written informed consent prior to study enrollment.

### Participants

Participants were recruited between September 2019 and November 2021. Inclusion criteria were (1) age between 18 and 80 years, (2) a subacute traumatic injury (< 28 weeks after injury, *sSCI*), or a chronic traumatic injury (> 2 years after injury, *cSCI*) with complete injury state (AIS A), a lesion level between Th1 and Th12 and (3) the ability to undergo MRI. Exclusion criteria included the presence of a cardiac pace maker, neurological disorders other than the spinal injury, depression, central nervous system tumors, severe traumatic brain injury (residues of micro bleeds on previous clinical brain MR imaging) or prior spinal surgery.

### Experimental design

#### Clinical assessments

Participants with SCI underwent a clinical evaluation (second-to-last author, with 25 years of experience) that included (a) the International Standards for Neurological Classification of Spinal Cord Injury (ISNCSCI) protocol for motor, light touch (LT), and pinprick (PP) scores^22^, (b) the Spinal Cord Independence Measure (SCIM) to measure daily life independence^23^, and (c) Hospital Anxiety and Depression Scale (HADS). The SCIM consists of three parts:(1) independence in self-care indoors/at home, (2) independence in bowel and bladder control and (3) indoor/outdoor mobility independence^24^.

#### MRI protocol

Following the minimum reporting standards for in vivo MRS (MRSinMRS)^25^ consensus recommendation, an overview of the study is available in Table 2.

**Table 2:** Study overview (consensus recommendation MRSinMRS).

1. Hardware	
(a) Filed strength [T]	3 T
(b) Manufacturer	Philips
(c) Model (software version)	5.4.1
(d) RF coils:	^1^H MRS, 32 channel head coil
(e) Additional hardware:	N/A
2. Acquisition	
(a) Pulse sequence	PRESS
(b) Volume of interest (VOI), locations	(1) pons, (2) cerebellar vermis, (3) cerebellar hemisphere
(c) Nominal VOI size [mm^3^]	(1) 25 × 20 × 20 mm^3^, (2) 20 × 10 × 20 mm^3^, (3) 20 × 10 × 20 mm^3^
(d) Repetition time (T_R_), echo time (T_E_)	T_R_ = 2000 ms, T_E_ = 30 ms
(e) Number of signal acquisitions (NSA)	(1) NSA = 128, (2) NSA = 256, (3) NSA = 128
(f) Additional sequence parameters	Metabolite cycled inversion pulse shift 180 Hz
(g) Water suppression method	Non-water suppressed metabolite cycling
(h) Shimming method	2nd order pencil-beam B_0_ field shimming
(i) Triggering or motion correction	N/A
3. Data analysis methods and outputs	
(a) Analysis software	^1^H MR spectroscopy data were processed from data/list format from the scanner using in-house developed MatLab scripts using Gannet (3.1) and MR libs software
(b) Processing steps deviating	Residual water suppression (HLSVD) and apodization filter (BW = 1 Hz) was applied to the non-water suppressed spectra before quantification by LCModel 6.3
(c) Output measure	Concentrations to internal water
4. Data quality	
(a) Reported values (SNR, linewidth)	SNR (as reported by LCModel)
	(1) cSCI: 23.1 ± 2.3 (19–26), sSCI: 23.0 ± 1.8 (21–26), HC: 25.9 ± 4.1 (20–34); overall: 24.2 ± 3.4 (19–34)
	(2) cSCI: 17.4 ± 1.6 (14–20), sSCI: 18.4 ± 0.8 (18–20), HC: 18.8 ± 2.5 (14–23); overall: 18.1 ± 2.0 (14–23)
	(3) cSCI: 12.9 ± 2.0 (10–18), sSCI: 13.4 ± 1.5 (12–16), HC: 14.8 ± 3.0 (10–20), overall: 13.7 ± 2.6 (10–20)
	FWHM (Hz)
	(1) cSCI: 7.4 ± 1.3 (4.8–9.7), sSCI: 6.2 ± 1.5 (4.8–8.8), HC: 6.7 ± 1.5 (4.8–9.7), overall: 6.9 ± 1.5 (4.8–9.7)
	(2) cSCI: 5.6 ± 0.9 (3.9–6.7), sSCI: 5.0 ± 0.4 (4.8–5.8), HC: 4.7 ± 1.0 (3.96.9), overall: 5.2 ± 1.0 (3.9–6.7)
	(3) cSCI: 6.5 ± 1.5 (4.8–11.7), sSCI: 5.8 ± 1.1 (4.8–7.8), HC: 6.5 ± 1.3 (4.8–9.7), overall: 6.4 ± 1.4 (4.8–11.7)
	No subjects excluded due to MRS data quality (two subjects excluded before the analysis due to high depression and anxiety score)
(b) Data exclusion criteria	
(c) Quality of measures of postprocessing model fitting	CRLB of NAA
	(1) cSCI: 3.1 ± 0.5 (2–4), sSCI: 2.6 ± 0.5 (2–3), HC: 2.7 ± 0.6 (2–4), overall: 2.9 ± 0.6 (2–4)
	(2) cSCI: 3.6 ± 0.5 (3–4), sSCI: 3.4 ± 0.5 (3–4), HC: 3.3 ± 0.5 (3–4), overall: 3.4 ± 0.5 (3–4)
	(3) cSCI: 4.7 ± 1.0 (3–6), sSCI: 4.2 ± 0.4 (4–5), HC: 4.3 ± 0.6 (3–5), overall: 4.4 ± 0.8 (3–6)
(d) sample spectrum	Figure 1

The study was performed with a 3.0 T MRI unit (Philips Achieva, release: 5.4.1; Philips Healthcare) using a 32-channel head coil (Philips Healthcare) to acquire spectra from three regions of interest in the brainstem and cerebellum, i.e. the pons, the cerebellar vermis and cerebellar hemisphere (Fig. 1).

MR measurement sequences included a survey acquisition, anatomic acquisitions (T1w, T2w, and FLAIR), and spectroscopic measurements (total duration of examination: 45 min).

We used the MC^26,27^ PRESS technique (TR/TE: 2000/30, spectral bandwidth: 2000 Hz, readout duration: 512 ms). The MRS sequences included a non-water suppressed reference shim scan (i.e. the water reference signal) and a MC sequence (i.e. the metabolite signal with alternative cycled inversion pulse) including higher-order shimming, broadband outer volume suppression and inner volume saturation pulses to minimize the chemical shift displacement artefact as described previously^6,27^.

We used the T1w (3D gradient echo, FOV = 320 × 260 × 192 mm^3^, slice thickness = 1 mm, repetition time (TR) = 7.6 ms, echo time (TE) = 3.6 ms, flip angle = 9°, standard sensitivity encoding reduction factor = 2), and T2w (3D spin echo, FOV = 250 × 250 × 180 mm^3^, slice thickness = 1 mm, TR/TE = 2500/247 ms, compressed sensitivity encoding reduction factor = 6) images to place the spectroscopic volume of interest. The fluid-attenuated inversion recovery (FLAIR, 3D inversion recovery, FOV = 250 × 250 × 180 mm^3^, slice thickness = 0.5 mm, TR/TE = 4800/1650 ms, compressed sensitivity encoding reduction factor = 10) was used to scrutinize for any elevated signal abnormalities and inhomogeneities of the parenchyma. The regions of interest (ROI) included (a) the pons (number of signal averages (NSA) = 128), (b) cerebellar vermis (NSA = 256) and (c) right hemisphere (NSA = 128). The dimensions were (a) 25 × 20 × 20 mm^3^ (10 mL), (b) and (c): 20 × 10 × 20 mm^3^ (4 mL).

### MRI postprocessing

#### Post-processing and metabolites quantification

We processed spectroscopic data in MatLab 2020a (9.8, MathWorks, Inc.) with an automated custom written post-processing pipeline incorporating Gannet^28^ (ver 3.1) and MR libs (https://github.com/chenkonturek/MRS_MRI_libs) software parts. We reconstructed the non-water-suppressed frequency-aligned measurement series as described previously^6,27^ and applied Hankel-Lanczos singular value decomposition (HLSVD) residual water suppression and apodization filter (BW = 1 Hz) before quantification by LCModel^29^ (ver 6.3–1N). Metabolite concentration values are referenced to the voxel’s internal water concentration taking into consideration a full tissue and relaxation correction according to Gasparovic et al^30,31^.

The basis set for spectral fitting included the following metabolites: N-acetyl-aspartate and N-acetyl-aspartyl-glutamate (NAA + NAAG = tNAA), GSH, glutamate and glutamine (Glu + Gln = Glx), glycerophosphochline and phosphocholine (GPC + PCh = tCho), Cr, scyllo-Inositol (sI) and myo-Inositol (mI). Metabolites were included based on the MRS consensus recommendations^32,33^. All MR spectroscopy acquisitions were performed by a trained spectroscopist (last author with 8 years of experience). Data analysis were performed by using an automated post-processing pipeline.

### Statistical analysis

We used R software (version 4.0.2, R Core Team 2020) with the tidyverse package^34^. Boxplots show data including medians and quartiles; medians and ranges are reported in the text. Group differences were assessed by using Kruskal–Wallis tests followed by Dunn’s test for pairwise multiple comparisons. Correlation analyses were performed using Spearman rank correlation. Linear regression model included clinical scores and metabolite concentration measurements. The confidence interval was set to 95% and *p* < 0.05 was assumed to indicate a significant difference. Age was included in the linear regression models as a covariate. Due to the exploratory nature of the study, no adjustments for multiplicity were applied. We included Bayesian statistics to quantify the evidence of the null hypothesis and the alternative hypothesis. We calculated the Bayes factor BF_10_ reflecting the support of the alternative hypothesis over the null hypotheses with the R package bayestestR (version 0.13.0)^35^.

## Supplementary Information

Supplementary Information.

## Data Availability

The data that support the findings of this study are available from the corresponding author upon reasonable request.
