# Supplementary material for: Metabolic profile of complete spinal cord injury in pons and cerebellum: A 3T 1H MRS study
Source: Sci Rep. 2023 May 4;13:7245. doi: 10.1038/s41598-023-34326-1 (PMC10160051; doi:10.1038/s41598-023-34326-1)
Supplement: Supplementary file 1 — Supplementary Information. [file 41598_2023_34326_MOESM1_ESM.docx]

**Supplementary Figure S1: Plots of non-significant Group Differences**

| Plots of non-significant group differences in the pons (A, B, C), cerebellar vermis (D, E, F), and cerebellar hemisphere (G, H, I) areas. The results of the Kruskal-Wallis tests with post-hoc Dunn’s test is reported.  cSCI: people with chronic SCI; GSH: glutathione; HC: healthy controls; SCI: spinal cord injury; sSCI: people with subacute SCI; tCr: total creatine; tCho: total choline containing compounds; tNAA: total N-acetyl-aspartate;  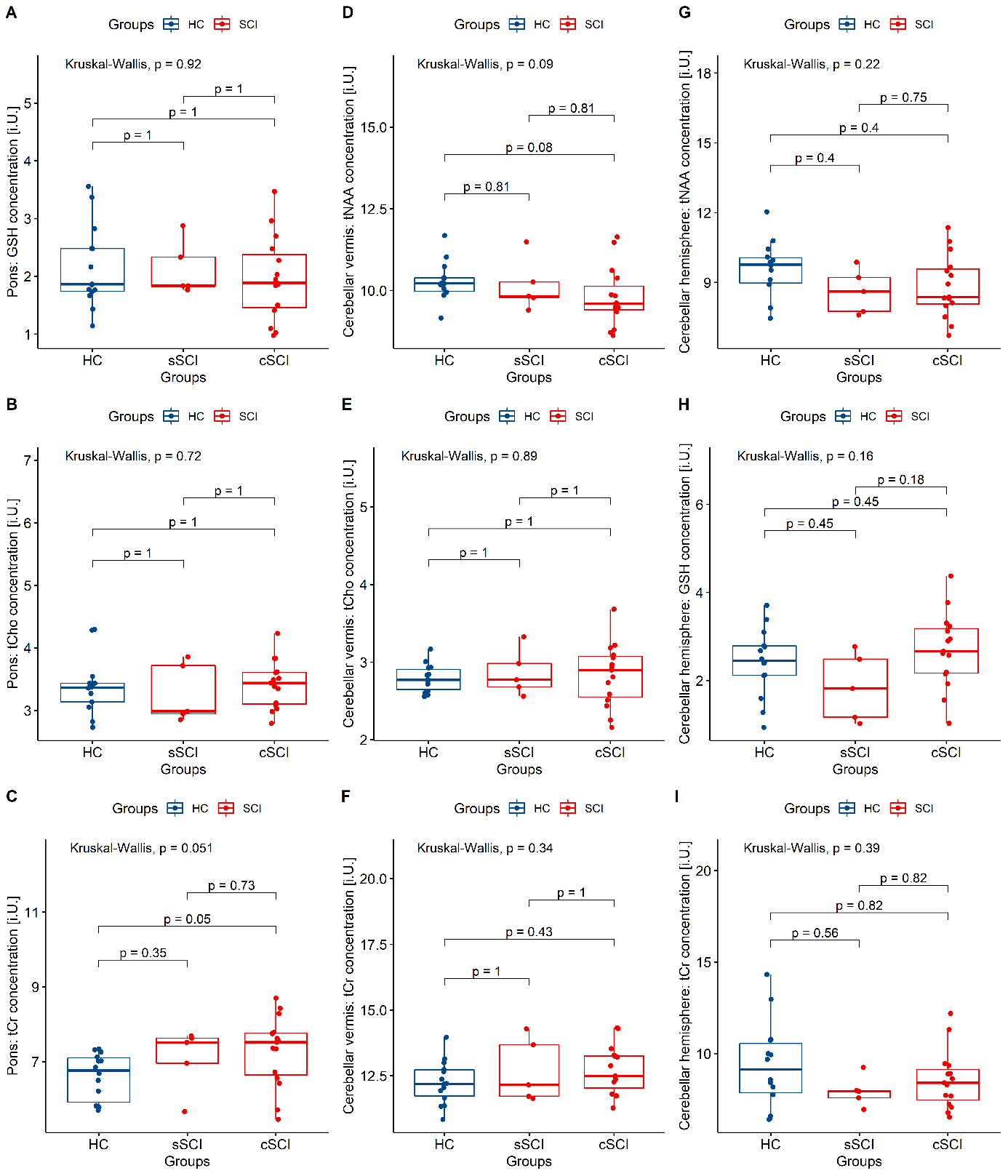 |
| --- |

**Supplementary Table S1: Group comparison statistics**

| Group differences between Healthy controls (HC), people with chronic SCI (cSCI), and people with subacute SCI (sSCI) for the metabolites total N-Acetyl-Aspartate (tNAA), glutathione (GSH), total choline containing compounds (tCho), and total creatine and phosphocreatine (tCr) in the three regions of interest.  *a) Kruskal-Wallis test (Degrees of freedom = 2)* | | | | | | | | | | | | | | | | | |
| --- | --- | --- | --- | --- | --- | --- | --- | --- | --- | --- | --- | --- | --- | --- | --- | --- | --- |
| Pons | | | | | | Cerebellar Vermis | | | | | | Cerebellar Hemisphere | | | | | |
| *Met* | Chi^2^ | | P_value_ | BF_10_ | | *Met* | Chi^2^ | | P_value_ | | BF_10_ | *Met* | | Chi^2^ | P_value_ | | BF_10_ |
| ***tNAA*** | 6.3 | | **0.04** | **1.42** | | *tNAA* | 4.8 | | 0.09 | | 0.60 | *tNAA* | | 3.0 | 0.22 | | 0.61 |
| *GSH* | 0.2 | | 0.92 | 0.26 | | ***GSH*** | 7.9 | | **0.02** | | **1.12** | *GSH* | | 3.7 | 0.16 | | 0.69 |
| *tCho* | 0.6 | | 0.73 | 0.24 | | *tCho* | 0.2 | | 0.89 | | 0.23 | ***tCho*** | | 9.9 | **0.01** | | **12.76** |
| *tCr* | 6.0 | | 0.051 | 1.28 | | *tCr* | 2.1 | | 0.34 | | 0.45 | *tCr* | | 1.9 | 0.39 | | 0.45 |
|  | | | | | | | | | | | | | | | | | |
| *b) Post-hoc Dunn’s test for tNAA (Pons), GSH (Cerebellar Vermis) and tCho (Cerebellar Hemisphere) for HC, cSCI, and sSCI* | | | | | | | | | | | | | | | | | |
| Met/Area | | Group 1 | | | Group 2 | | | Statistics | | P_value_ | | | Adj. P_value_ | | | BF_10_ | |
| tNAA in pons | | HC | | | sSCI | | | -0.38 | | 0.70 | | | 0.70 | | | 0.45 | |
|  |  | ***HC*** | | | ***cSCI*** | | | -2.45 | | 0.01 | | | **0.04** | | | **2.15** | |
|  |  | sSCI | | | cSCI | | | -1.37 | | 0.17 | | | 0.34 | | | 1.28 | |
| GSH in cerebellar vermis | | HC | | | sSCI | | | -0.28 | | 0.78 | | | 0.78 | | | 0.51 | |
|  |  | ***HC*** | | | ***cSCI*** | | | 2.49 | | 0.01 | | | **0.04** | | | **1.19** | |
|  |  | sSCI | | | cSCI | | | 2.07 | | 0.04 | | | 0.08 | | | 1.06 | |
| tCho in Cerebellar Hemisphere | | ***HC*** | | | ***sSCI*** | | | -2.64 | | 0.01 | | | **0.02** | | | **4.46** | |
|  |  | ***HC*** | | | ***cSCI*** | | | -2.57 | | 0.01 | | | **0.02** | | | **7.24** | |
|  |  | sSCI | | | cSCI | | | 0.82 | | 0.4 | | | 0.4 | | | 0.57 | |
| Note: Adj. P_value_= adjusted P value; BF_10_=Bayes Factor; Chi^2^=Chi-Square statistics; Met=metabolite; GSH=Glutathione, tCho= total choline containing compounds, tCr= total creatine and phosophocreatine, tNAA=total N-acetyl-aspartate and NAA-Glutamate | | | | | | | | | | | | | | | | | |

**Supplementary Table S2: Correlation statistics**

| Correlation analysis in people with SCI for the metabolites (total N-Acetyl-Aspartate (tNAA), glutathione (GSH), total choline containing compounds (tCho), and total creatine and phosphocreatine (tCr)), and the clinical scores (light touch (LT), pinprick(PP), and spinal cord independance measure (SCIM)) in three regions of interest. | | | | | |
| --- | --- | --- | --- | --- | --- |
| a) Pons | | | | | |
| Clin.Score vs. Met. | tNAA | GSH | tCho | tCr | tNAA/tCr |
| LT_tot_ | rho = 0.337  p = 0.15  BF_10_ = 0.66 | rho = 0.023  p = 0.92  BF_10_ = 0.48 | ***rho = - 0.52***  ***p = 0.02***  ***BF_10_ = 3.32*** | rho = - 0.43  p = 0.06  BF_10_ = 1.20 | ***rho = 0.549***  ***p = 0.01***  ***BF_10_ = 2.60*** |
| PP_tot_ | rho = 0.300  p = 0.20  BF_10_ = 0.64 | rho = 0.049  p = 0.84  BF_10_ = 0.48 | ***rho = - 0.55***  ***p = 0.01***  ***BF_10_ = 3.23*** | rho = -0.45  p = 0.05  BF_10_ = 1.19 | ***rho = 0.542***  ***p = 0.01***  ***BF_10_ =2.37*** |
| SCIM_tot_ | rho = -0.14  p = 0.56  BF_10_ = 0.62 | rho = 0.188  p = 0.43  BF_10_ =0.51 | rho = 0.009  p = 0.97  BF_10_ = 0.48 | rho = -0.06  p = 0.81  BF_10_ = 0.48 | rho = 0.013  p = 0.96  BF_10_ = 0.48 |
| SCIM 1 | rho = -0.15  p = 0.516  BF_10_ = 0.84 | rho = 0.072  p = 0.76  BF_10_ = 0.47 | rho = - 0.19  p = 0.43  BF_10_ = 0.56 | rho = 0.118  p = 0.62  BF_10_ = 0.49 | rho = -0.20  p = 0.40  BF_10_ = 0.48 |
| SCIM 2 | rho = -0.01  p = 0.96  BF_10_ = 0.48 | rho = 0.206  p = 0.38  BF_10_ = 0.64 | rho = 0.008  p = 0.97  BF_10_ = 0.50 | rho = -0.02  p = 0.94  BF_10_ = 0.48 | rho = 0.094  p = 0.70  BF_10_ =0.48 |
| SCIM 3a | rho = -0.23  p = 0.33  BF_10_ = 1.98 | rho = 0.051  p = 0.83  BF_10_ = 0.49 | rho = 0.104  p = 0.66  BF_10_ = 0.56 | rho = 0.178  p = 0.45  BF_10_ =0.48 | rho = -0.27  p = 0.25  BF_10_ =0.70 |
| b) Cerebellar Vermis | | | | | |
| Clin.Score vs. Met. | tNAA | GSH | tCho | tCr | tNAA/tCr |
| LT_tot_ | rho = 0.326  p = 0.16  BF_10_ =0.69 | rho = -0.28  p = 0.24  BF_10_ = 0.61 | rho = -0.11  p = 0.65  BF_10_ = 0.48 | **rho = -0.47**  **p = 0.04**  **BF_10_ = 1.61** | **rho = 0.620**  **p = 0.004**  **BF_10_ = 11.39** |
| PP_tot_ | rho = 0.320  p = 0.17  BF_10_ = 0.65 | rho = -0.24  p = 0.31  BF_10_ = 0.57 | rho = -0.16  p = 0.50  BF_10_ = 0.48 | **rho = -0.48**  **p = 0.03**  **BF_10_ = 1.72** | **rho = 0.608**  **p = 0.004**  **BF_10_ =10.3** |
| SCIM_tot_ | rho = -0.07  p = 0.78  BF_10_ = 0.49 | rho = 0.299  p = 0.20  BF_10_ = 0.77 | rho = 0.048  p = 0.84  BF_10_ = 0.49 | rho = 0.179  p = 0.45  BF_10_ = 0.63 | rho = -0.17  p = 0.46  BF_10_ = 0.53 |
| SCIM part 1 | rho = -0.04  p = 0.87  BF_10_ = 0.48 | rho =0.158  p = 0.51  BF_10_ = 0.64 | rho = -0.37  p =0.11  BF_10_ = 0.75 | rho =0.364  p = 0.12  BF_10_ = 1.01 | rho = -0.36  p = 0.12  BF_10_ = 0.76 |
| SCIM part 2 | rho = 0.075  p =0.75  BF_10_ = 0.55 | rho = 0.161  p =0.50  BF_10_ = 0.55 | rho = 0.326  p = 0.16  BF_10_ = 0.83 | rho = 0.155  p = 0.51  BF_10_ = 0.53 | rho = 0.015  p = 0.95  BF_10_ = 0.48 |
| SCIM part 3a | rho =-0.15  p = 0.54  BF_10_ = 0.51 | rho = 0.455  p = 0.05  BF_10_ = 1.93 | rho = - 0.25  p = 0.29  BF_10_ = 0.53 | rho = 0.171  p = 0.47  BF_10_ = 0.53 | rho =-0.18  p = 0.44  BF_10_ = 0.74 |
| c) Cerebellar Hemisphere | | | | | |
| Clin.Score vs. Met. | tNAA | GSH | tCho | tCr | tNAA/tCr |
| LT_tot_ | rho = -0.06  p = 0.79  BF_10_ = 0.52 | rho = -0.21  p = 0.36  BF_10_ = 0.57 | rho =-.026  p = 0.26  BF_10_ = 0.69 | rho = -0.07  p = 0.77  BF_10_ = 0.48 | rho = -0.01  p = 0.99  BF_10_ = 0.48 |
| PP_tot_ | rho = -0.09  p = 0.71  BF_10_ = 0.56 | rho = -0.18  p = 0.46  BF_10_ = 0.55 | rho =-0.28  p = 0.23  BF_10_ = 0.70 | rho = -0.01  p = 0.97  BF_10_ = 0.47 | rho = -0.05  p = 0.83  BF_10_ = 0.50 |
| SCIM_tot_ | rho =0.224  p = 0.34  BF_10_ = 0.69 | ***rho = 0.555***  ***p = 0.01***  ***BF_10_ = 4.66*** | rho = 0.094  p = 0.69  BF_10_ = 0.48 | rho = -0.29  p = 0.22  BF_10_ = 1.33 | rho =0.377  p = 0.10  BF_10_ = 1.69 |
| SCIM 1 | rho = -0.06  p = 0.79  BF_10_ = 0.49 | ***rho = 0.661***  ***p = 0.002***  ***BF_10_ = 15.0*** | rho = -0.08  p = 0.72  BF_10_ = 0.58 | rho = -0.05  p = 0.82  BF_10_ = 0.81 | rho = 0.090  p = 0.71  BF_10_ = 0.65 |
| SCIM 2 | rho = 0.273  p = 0.24  BF_10_ = 0.89 | rho = 0.288  p = 0.22  BF_10_ = 1.03 | rho = 0.047  p = 0.84  BF_10_ = 0.48 | **rho = -0.46**  **p = 0.04**  **BF_10_ =2.41** | **rho = 0.533**  **p = 0.02**  **BF_10_ =4.22** |
| SCIM 3a | rho = 0.203  p = 0.39  BF_10_ = 0.57 | ***rho = 0.537***  ***p = 0.02***  ***BF_10_ = 9.64*** | rho = 0.005  p = 0.98  BF_10_ = 0.50 | rho = -0.14  p = 0.56  BF_10_ = 0.53 | rho = 0.263  p = 0.26  BF_10_ = 0.63 |
